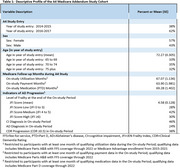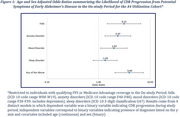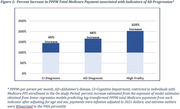# Alzheimer's Disease (AD) Progression and Healthcare Resource Utilization (HRU) in Preclinical AD

**DOI:** 10.1002/alz70860_103138

**Published:** 2025-12-23

**Authors:** Julie Chandler, Zachary Sheff, Nalin Payakachat, Roy Yaari, Karen Chilcott Holdridge, Joanna Kubisiak, Angelina Lee, Reisa A. Sperling, Paul S. Aisen, Michael S. Rafii

**Affiliations:** ^1^ Eli Lilly & Company, Indianapolis, IN, USA; ^2^ Westat Inc., Rockville, MD, USA; ^3^ Harvard Medical School, Boston, MA, USA; ^4^ Center for Alzheimer Research and Treatment, Department of Neurology, Brigham and Women's Hospital, Boston, MA, USA; ^5^ Alzheimer's Therapeutic Research Institute, Keck School of Medicine, University of Southern California, San Diego, CA, USA

## Abstract

**Background:**

Medical diagnoses and HRU in preclinical AD are not well characterized and may reveal indicators of clinical progression in the asymptomatic stages of AD. This study describes AD progression and HRU among cognitively unimpaired individuals with elevated amyloid.

**Methods:**

An addendum to the Anti‐Amyloid Treatment in Asymptomatic AD (A4) clinical trial followed 246 Medicare beneficiaries who consented to have their Medicare claims linked to study outcomes. A4 study outcomes, including baseline demographics and the Clinical Dementia Rating Scale‐Global Score (CDR‐GS), were linked with diagnoses, encounters by setting, payments, medications, and the JEN Frailty Index (JFI, a claims‐based indicator of functional impairment) from the Medicare data to evaluate AD progression from A4 randomization to last study visit. From the A4 Study, AD progression was defined as two consecutive, or one final, CDR‐GS score(s) ≥ 0.5. Within the Medicare data, three measures of AD progression were defined: cognitive impairment (CI) diagnosis, AD diagnosis, and JFI ≥ 6. Associations between AD progression and presence of disease and encounter variables were evaluated with odds ratios; linear regression evaluated payments.

**Results:**

Average follow‐up months for utilization (diagnoses and encounters), payments, and medications were 67, 64, and 69, respectively; 38% of participants had CDR‐GS progression in A4; within the Medicare data, 46% had a CI diagnosis, 24% an AD diagnosis, and 30% JFI ≥ 6 (Table 1). CDR‐GS progression was associated with diagnoses of mood disorders (OR: 1.97; 95% CI: 1.08‐3.59), anxiety disorders (OR: 2.37; 95% CI: 1.34‐4.21), and falls (OR: 3.12; 95% CI: 1.47‐6.59; Figure 1). AD diagnosis was associated with the highest quartile of inpatient utilization (OR: 2.59; 95% CI: 1.35‐5.00), as was JFI ≥ 6 (OR: 2.74; 95% CI: 1.46‐5.14). Payments increased with AD progression defined by CI diagnosis (45% increased payments, *p* = 0.0351), AD diagnosis (66% increase, *p* = 0.0105), and JFI ≥ 6 (103% increase, *p* = 0.0003), but not with CDR‐GS progression (Figure 2).

**Conclusions:**

As AD progresses from preclinical to early symptomatic, Medicare spending increases. These results highlight the need for additional research on cost savings associated with delaying progression to symptomatic stages of disease.